# Cognition, Emotion and Behavior in Children with Tourette’s Syndrome and Children with ADHD-Combined Subtype—A Two-Year Follow-Up Study

**DOI:** 10.1371/journal.pone.0144874

**Published:** 2015-12-16

**Authors:** Kjell Tore Hovik, Kerstin J. Plessen, Andrea E. Cavanna, Erik Winther Skogli, Per Normann Andersen, Merete Øie

**Affiliations:** 1 Innlandet Hospital Trust, Division Mental Health Care, Lillehammer, Norway; 2 University of Oslo, Institute of Psychology, Oslo, Norway; 3 Child and Adolescent Mental Health Centre, Capital Region Psychiatry, University of Copenhagen, Copenhagen, Denmark; 4 Institute for Clinical Medicine, University of Copenhagen, Copenhagen, Denmark; 5 Department of Neuropsychiatry, University of Birmingham and BSMHFT, Birmingham, United Kingdom; 6 School of Life and Health Sciences, Aston University, Birmingham, United Kingdom; 7 Sobell Department of Movement Disorders, Institute of Neurology, London, United Kingdom; 8 Lillehammer University College, Department of Education and Social Work, Lillehammer, Norway; Harvard Medical School, UNITED STATES

## Abstract

**Objective:**

This two-year follow-up study investigates the course of and association among measures of cognitive control, focused attention, decision-making and symptom severity (anxiety, depression and behavior) in children and adolescents with Tourette’s Syndrome (TS) or Attention-Deficit/Hyperactivity Disorder-Combined subtype (ADHD-C).

**Method:**

19 children with TS, 33 with ADHD-C, and 50 typically developing children (TDC) were examined with a battery of psychometric measures and rating forms at baseline and two-years later.

**Results:**

All three groups improved likewise in measures of cognitive control over time, whereas only the TDC improved in focused attention. The group of children with TS with comorbidities performed more similar to the children with ADHD-C in cognitive control at T1 and T2, whereas the children with TS without comorbidities performed more similar to the TDC in cognitive control at T1 and T2. In the decision-making task, the children with TS (with or without comorbidities) preferred a safer strategy in selecting advantageous choices than the children with ADHD-C and the TDC at T2. Children with TS and children with ADHD-C showed higher symptoms of anxiety and depression and more problems with emotional control compared with TDC at both time points. Finally, children with ADHD-C self-reported more depression symptoms than those with TS at both assessments. For the TS group, safer decision-making was related to better emotional control, and this relationship was stronger for the TS subgroup without comorbidities.

**Conclusion:**

This study emphasizes the importance of addressing symptoms of anxiety and depression in children with TS or ADHD-C, identifying the effect of comorbidities in children with TS, and that children with TS or ADHD-C likely differ in their sensitivity to reinforcement contingencies.

## Introduction

Tourette’s Syndrome (TS) and Attention-Deficit/Hyperactivity Disorder (ADHD) are both frequent and co-occurring neurodevelopmental disorders associated with problems in regulating thoughts, emotion and behavior [1, 2]. Several studies have reported a genetic overlap within these disorders, however, the exact relationship is not clear [3, 4]. Children with TS or ADHD often show impaired performance compared with typically developing children (TDC) on neurocognitive tests requiring cognitive control [5, 6]. Cognitive control refers to top-down mechanisms that coordinate lower-level sensory, emotional and motor processes along an internal goal, and thus provide mechanisms for self-regulating behavior [7–9]. Most human behavior reflects the interplay of lower-level processes (bottom-up influences) and higher-level goals (top-down influences) [10, 11]. Focused attention plays a critical role in the control of cognition and emotion by activating brain regions (e.g. anterior cingulate cortex) involved in monitoring and resolving competing response alternatives [12, 13]. Decision-making tasks incorporate both cognitive and affective influence affecting behavior [14, 15]. Hence, the combination of control over both cognition and emotion plays an important role in the successful self-regulation of behavior [16, 17]. The current two-year, follow up study thus includes measures assessing various aspects of control over cognition and ratings of anxiety, depression and emotional control problems to gain knowledge of the development and interplay of these processes in children with TS and ADHD-C who display problems in several of these areas [18].

According to an influential theory, core cognitive control processes involve working memory, inhibition and mental flexibility [19]. Deficits in cognitive control have been reported in individuals with both TS and/or ADHD [5, 20, 21]. Impairments in cognitive control are deemed central to ADHD [22, 23], whereas the evidence for children or adolescents with TS is less clear [24]. Individuals with ADHD remain impaired in tasks of cognitive control, despite documented improvement throughout development [22, 25]. To our knowledge, no prior studies have investigated developmental changes in performance on measures of cognitive control in youth with TS compared with youth with ADHD-C or TDC.

Tasks measuring decision-making and motivation involve more affective salience than tests measuring cognitive control. Such tasks typically require a choice between competing alternatives involving risk for minimizing or reward for maximizing outcome [26]. The relationship between choices and outcomes in these tasks depends on a close interplay between brain regions mediating both cognition and emotion [14, 27, 28]. Notably, decision-making skills depend on maturational factors that are not fully developed until late adolescence or young adulthood [29]. A study examining decision-making in children with TS reported impaired performance compared with controls [20], whereas adults with TS did not show any deficits [30]. Children with ADHD often react differently to risk and reward than TDC [31]. Several studies administering a children’s version of a decision-making task found impairment in youth with ADHD [32–34], whereas two other studies did not report any decision-making deficit in youth with ADHD [35, 36]. The preference of children with ADHD for immediate over delayed reward and their reduced sensitivy to reinforcement influenced their response style compared with TDC in decision-making tasks [37, 38].

Children with TS or ADHD-C have been found to report rsignificantly higher rates of symptoms of anxiety and depression than TDC [39]. Anxiety is common in children with TS [24], and a recent 4-year follow-up study involving children with tics documented a significant increase in emotional symptoms from baseline to follow-up [40]. Up to 30 percent of children with ADHD suffer from depression [41], however, children with ADHD-C often report higher levels of anxiety and depression symptoms than children with ADHD-Inattentive subtype (ADHD-I) [42]. Depression symptoms in young persons with TS [40] or ADHD [41] commonly increase over time, and the presentation of these symptoms in youth in general is significantly related to the development of major depression in adulthood [43]. In TDC, symptoms of anxiety or depression are related to a reduction in cognitive control and an exacerbation of existing behavioral problems [16, 44]. A better understanding of the course of anxiety and depression symptoms in children with TS and ADHD-C may help to individualize clinical treatment of these two disorders.

Problems regulating their behavior in everyday settings characterize young persons with both ADHD or TS regardless of other comorbidity [18, 45–47]. However, children with the Combined subtype of ADHD (i.e. ADHD-C) display most behavioral difficulties of all ADHD subtypes [48]. Difficulties regulating behavior is most reliably present in assessments of how children perform over time in real-life situations where they must regulate their actions and responses on their own [49]. Parents are in an ideal situation to rate their child’s ability to appropriately adapt their behavior in everday life. For children with ADHD in which executive impairment is considered a central deficit, for example, cognitive control tasks administered in a laboratory setting revealed executive impairment in only about 50% [5]. In contrast, a recent study found that 79% of the parents of the youth with ADHD-C rated emotional control as a significant problem, and 94% of the parents of youth with TS rated emotional control as a significant problem, using a parent-rated questionnaire to describe their child’s everyday executive control [18]. The term *emotional control* here refers to the ability to adjust emotionally colored behavior (e.g. mood lability, agitation, excitement) appropriately in situations involving high affective salience [50]. To our knowledge, no studies to date have investigated the development of emotional control abilities in youth with TS or ADHD-C over time.

We thus set out to investigate the development of a) cognitive control, b) focused attention, c) symptoms of anxiety and depression, and d) emotional control problems in children with TS compared with ADHD-C and TDC over a two-year span. Possible problems in these areas at baseline and follow-up among the groups were also examined. Moreover, we wanted to examine how changes in cognitive performance relate to changes in self-reported anxiety or depression symptoms or parent-reported emotional control problems. Based on the available literature, we expected that children with TS or ADHD-C would improve similarly over two years to the TDC in a composite measure of cognitive control (working memory, inhibition and mental flexibility). Consistent with the results of a study from our research group involving a larger group of ADHD children not divided into subtypes, we expected that children with ADHD-C, but not children with TS, would show difficulties in cognitive control compared with TDC at T1 and T2. [25]. Earlier studies involving children with ADHD [33, 35, 36] and adults with TS [30] on a decision-making task similar to the one used in the current study have not shown differences in overall favorable outcome. We aimed to examine the hypothesis that due to different thresholds of sensitivity to reinforcement contingencies [37], children with TS and children with ADHD-C would prefer different choices offering the same overall outcome (safer versus riskier, respectively) in a decision-making task. Moreover, self-reported symptoms of anxiety and depression and parent-reported emotional control problems were expected to increase after two years in line with duration of illness in both clinical groups. Based on research indicating that cognitive control processes play a central role in regulating emotions and behavior [26], we expected that changes in measures of cognitive control would relate to changes in self-reports of anxiety and depression symptoms and parent-report of emotional control problems. Finally, we intended to investigate relationships between changes in performance on the decision-making task and changes in self-report of emotional symptoms and parent-report of emotional control problems after two years, because the decision-making task is more sensitive to emotionally salient task contingencies [29].

## Method

### Sample

We recruited 19 children with TS, 33 children with ADHD-C and 50 children with TDC for the study. The clinical sample was referred consecutively to the Child and Adolescent Mental Health Centres at Innlandet Hospital Trust (IHT) in Norway in 2009 and 2010. All participants underwent a diagnostic assessment based on separate interviews of the participant and parent(s) using the Schedule for Affective Disorders and Schizophrenia for School Age Children/Present and Lifetime version-2009 (K-SADS-PL) [51]. Experienced clinicians familiar with diagnosing children and adolescents with neuropsychiatric disorders conducted the interviews. The initial diagnostic evaluation was supplemented with information from the ADHD Rating Scale IV [52], the Child Behavior Checklist (CBCL) [53], the Autism Spectrum Screening Questionnaire [54], and the Yale Global Tic Severity Scale [55]. Additional information about academic and social functioning was included in the assessment. A diagnosis was confirmed if DSM-IV-TR criteria [56] were met through an evaluation of K-SADS-PL, parent reports and self-reporting together with information from teachers concerning academic and social functioning. TDC were recruited from local schools and underwent the same assessment procedure as the clinical participants. The TDC received a gift certificate worth USD 33 for their participation; their parents were not compensated for participating. Exclusion criteria for all groups were prematurity (< 36 weeks), IQ estimate below 70, and neurological disease. Additional criteria of exclusion for the TDC were any history of a psychiatric disorder, dyslexia or head injury. Comprehensive information regarding recruitment procedure and diagnostic measures is described elsewhere (e.g. [18, 36, 56]). No significant difference in age, gender or FSIQ was found for the groups at T1. At T2, the level of FSIQ was significantly lower for the ADHD-C group compared with the TDC, whereas no difference was registered between the two clinical groups (Table 1).

**Table 1:** Demographic characteristics for matched samples: means, standard deviations and ANOVAs of group and assessment time.

			Baseline (T1)					Follow-up (T2)		
Variable	TS ^a)^	ADHD-C ^b)^	TDC	Group comparisons		TS	ADHD-C	TDC	Group comparisons	
	(n = 19)	(*n =* 33)	(n = 50)	Chi-sq./ F	*p*	(n = 19)	(n = 33)	(n = 50)	Chi-sq./ F	*p*
Sex (m/f)	16/3	20/13	32/18	(2, 102) = 3,4	ns	16/3	20/13	32/18	(2, 102) = 3,4	ns
Age in months	147 (27)	144 (26)	144 (24)	(2,99) = .09	ns	171 (27)	168 (26)	169 (24)	(2,98) = .09	ns
FSIQ ^c)^	102 (15)	97 (14)	104 (13)	(2,99) = 2.6	ns	101 (13)	97 (14)	106 (13)	(2,93) = 5.3	<.01
Motor tics, *YGTSS* ^d)^	12.1 (7.3)	.33 (1.9)	0.0	(2,99) = 102.1	<.001	5.9 (5.0)	.73 (2.6)	.22 (.89)	(2,99) = 29.2	<.001
Phonic tics, *GTSS* ^d)^	10.2 (5.2)	.06 (.35)	0.0	(2,99) = 159.6	<.001	5.4 (5.8)	.99 (3.0)	.22 (.89)	(2,99) = 20.2	<.001
ADHD symptoms^e)^	25.6 (14)	29.9 (9.8)	2.6 (3.0)	(2,99) = 118.5	<.001	15.9 (10)	21.0 (12)	2.2 (2.7)	(2,98) = 54.6	<.001

Note. Tourette’s Syndrome (TS); Typically developing children (TDC); Attention/deficit Hyperactivity Disorder, Combined type (ADHD-C).

^a)^ At baseline (T1), 11 patients with TS had comorbid disorders: 1xObsessive Compulsive Disorder (OCD), 1xOppositional Defiant Disorder (ODD), 1x ODD & ADHD-C, 2xADHD-I, 2xADHD-C, 3xAsperger’s syndrome, 1xADHD-I/Asperger’s syndrome. Two received a low dose of Quetiapine and two received a low dose of Aripiprazole, whereas the remaining fifteen participants with TS were medicine naïve upon inclusion and testing. At T2, 7 patients in the TS group no longer satisfied formal diagnostic criteria for a tic disorder; 1 fulfilled criteria for Chronic Motor Tic Disorder. At T2, the two children with TS and either OCD or ODD retained this comorbid diagnosis at T2. One child with TS and no comorbid diagnosis at T1 fulfilled criteria for a comorbid general anxiety disorder at T2.

^b)^ At T1, only two children with ADHD were on any medication, with low doses of Risperidone and Quetiapine, respectively. At T2, 11 retained the diagnosis of ADHD-C; 6 fulfilled criteria for ADHD-I, and 2 no longer fulfilled criteria for ADHD. No other co-occurring disorders were registered in this group.

^c)^ Full scale IQ (FSIQ). IQ estimated measures from the Wechsler Abbreviated Scale of Intelligence (WASI).

^d)^ Yale Global Tic Severity Scale (YGTSS). The group with TS had significantly more motor and phonic tics than the group with ADHD-C and TDC at T1 and T2.

^e)^ ADHD Rating Scale IV—Total Score. The children with ADHD-C had significantly more ADHD symptoms than TS at T1 and T2, and the children with TS had significantly more ADHD symptoms than the TDC at T1 and T2.

None of the participants in the study met criteria for a major depressive episode (Table 1). The rate of co-occurring conditions and gender balance in the TS group is consistent with other clinical samples [57]. The same participants were followed up at T1 and T2 with no dropouts. Identical assessment procedures were used at T1 and T2 [18, 58]. The same parent or parents interviewed at T1 (most commonly the mother) for each participant was invited for the interview at T2. At T2, eight of the children with TS at T1 did not fill the criteria required for motor or phonic tics to receive a TS diagnosis (42%).

#### Treatment from T1 to T2

At the time of referral and first assessment, T1, none of the participants were medicated with a psychostimulant. After being assessed at T1, participants in the clinical groups received treatment-as-usual for their disorder at their respective outpatient clinics (Table 1). After being diagnosed at T1, a total of 58% of the children with ADHD and 32% of the children with TS received psychostimulant treatment during the interim period. With the exception of one female participant with ADHD-C, however, all participants treated with psychostimulants discontinued their use at least 24 hours prior to the neurocognitive assessments at T2.

### Measures

#### Measures of cognitive control

**Focused Attention: Variability of Standard Error:** In the Conners’ Continuous Performance Test-II (CCPT-II), a repetitive array of visual stimuli on a computer screen is presented to the test-participant for 14 minutes [59]. The measure Variability of Standard Error is a measure of response speed consistency across 18 separate segments of the test. A higher score on this measure is an indication of a greater inconsistency in response speed during the task. In a study using principal component analysis to examine various aspects of attention, the Variability of Standard Error measure related in particular to the ability to focus attention on task [60].

**Composite measure of cognitive control:** The raw scores of three measures of core cognitive control (working memory, inhibition, and mental flexibility) were converted into scaled scores based on the average performance of the TDC at T1. Two of the measures (inhibition and mental flexibility) were reversed before aggregating the results into a composite score, so that a higher score for all measures indicates better performance. The three measures included in the composite are described below:

**Working memory: Letter-number sequencing test (LNS) [61]:** The LNS consists of ten items, each containing three trials with the same number of different combinations of digits and letters. Following a verbal presentation, the participant must recall the numbers in ascending order and the letters in alphabetical order (Wechsler, 2004). In the current study, total correct recalled trials were examined.

**Inhibition: Color-Word Interference test, Condition 3 (CW3) [62]:** In this test, the participant must inhibit the automatic response to read the printed word of a color and instead name the dissonant ink-color. The raw score of completion time in seconds and the number of errors were included.

**Mental flexibility: Color-Word Interference test, Condition 4 (CW4) [62]:** In CW4, the test-participant must switch between reading printed words of colors and name the dissonant ink color. Completion time in seconds was measured.

#### Measures of decision-making

**Decision-making: The Hungry Donkey Task (HDT):** The computer-based HDT [63] is a children’s version of the Iowa Gambling Task (IGT) [64]. The basic format of the IGT (gambling) is retained, but the HDT (a pro-social game) is considered a more appropriate decision-making task for children [63]. Participants help a hungry donkey to collect as many apples as possible by freely choosing among four doors (A, B, C, or D). The net win and loss varies among the choice of doors, and overall gain/loss is displayed with a red/green bar at the bottom of the screen. Doors A and B represent disadvantageous choices (resulting in overall loss), and Doors C and D represent advantageous choices (resulting in overall gain). The selection of Doors A and C involve frequent, but lower-level losses, whereas the selection of Doors B and D involve infrequent, but higher-level losses. The task ends after completing 10 blocks involving 150 individual trials. As the risk parameters are uncertain at the start of the task, early choices are considered decision-making under ambiguity, whereas later choices are considered to be decision-making under risk [65]. We termed results for the last four blocks of Door C a “Safer Choice”, based on the logic that by selecting this door the subject ensures a steady gain in outcome by having to endure regularly occurring low-level losses. We termed results for the last four blocks of Door D a “Riskier Choice”, because, although it offers the same overall gain as Door C, the subject must endure sudden, large losses. The two advantageous doors thus offer alternative “gain versus pain” schedules and ratios. For a detailed account of the HDT, see Crone & van der Molen [63].

#### Symptoms of anxiety and depression—self-report

The Revised Children’s Manifest Anxiety Scale, second edition (RCMAS-2) [66] is a self-report instrument designed to measure anxiety symptoms in children 6 to 19 years of age. Children respond either “Yes” or “No” to 49 items. Three factors of anxiety are assessed: Physiological Anxiety, Worry and Social Anxiety. Elevated raw-scores indicate a higher degree of anxiety symptoms, and raw-scores were used in the analyses. The RCMAS has satisfactory psychometric properties with high test-retest reliability [67, 68], and consistent construct validity [69–72]. Satisfactory psychometric properties have also been replicated in other cultures [68, 73–75].

The Short Mood and Feelings Questionnaire (SMFQ) [76] is designed to measure symptoms of depression in children 8 to 18 years of age. The short version consisting of 13 items is derived from the original 30-item Mood and Feelings Questionnaire (MFQ) [77], which has been shown to identify major depressive episodes and other mood disorders in youth diverse in demographic and clinical characteristics [78]. Children self-report symptoms according to a three-point scale (“not true”, “sometimes true” and “true”). Elevated raw-scores indicate a higher degree of depression symptoms, and the raw-scores were used in the analyses. The SMFQ has demonstrated high internal consistency (Crohnbach’s alpha = .90) [79], and test-retest stability in children [77]. SMFQ correlates strongly with the Children’s Depression Inventory (CDI) [80] and the depression score in the Diagnostic Interview Schedule for Children (DISC-C) (r = .67 and .51, respectively) [77].

#### Emotional control in everyday settings—parent-rating

The Behavior Rating Inventory of Executive Function (BRIEF) is a questionnaire that measures self-regulatory abilities needed for adaptive functioning in everyday situations for children aged 5 to 18 [50]. Parent-rated instruments are highly sensitive to the problems young patients experience in everyday life and may be a better predictor of real-world difficulties than the evaluation of cognitive control problems using neuropsychological tasks [81, 82]. The BRIEF is composed of eight clinical scales (Inhibition, Shift, Emotional Control, Initiate, Working Memory, Plan/Organize, Organization of Materials, and Monitor). High internal consistency (Chronbach’s α = .76-.92) has been reported for the Norwegian version of the parent-rating form [83]. In the current study, the Emotional Control scale was used to assess the control of more emotionally salient everyday behavior (i.e. emotional control) [84], with elevated T-scores on this measure indicating a higher degree of impairment.

### Data Analyses

Data analyses were conducted using the statistical package SPSS for Windows, version 21.0 (IBM, SPSS, Inc., Chicago, IL). Demographic characteristics were examined using the chi-square test for independence (nominal variables) and one-way analysis of variance (ANOVA; continuous variables) followed up by post hoc tests for all group comparisons. Because of multiple comparisons, Bonferroni-Holm corrections were used to control for chance findings by reducing the global alpha level (α = .05) proportionately to the number of comparisons being performed [25, 85]. Mixed between-within subjects ANOVAS (mixed ANOVA) were conducted for each dependent variable to estimate the effect of time and group on performance. Significant interactions were followed-up with post-doc tests and repeated measures ANOVAS for each group. Separate ANOVAs were conducted to examine relative task performance or symptom load between groups at T1 and T2. Non-parametric analyses applying the Kruskal-Wallis test (alpha level of .01) were also conducted for the analyses relating to symptomatology. Correlation analyses (Pearson) were used to investigate associations between changes in measures assessing focused attention, core cognitive control (working memory, inhibition, mental flexibility) and decision-making between T1 and T2, and changes in symptoms of anxiety and depression and emotional control problems. Full Scale Intelligence Quotient (FSIQ) was used as a covariate in the analyses of measures assessing focused attention, cognitive control and decision-making. Motor tics, phonic tics and ADHD symptoms were used as covariates in the analyses of emotional symptoms (emotional control problems, symptoms of anxiety, and symptoms of depression) to account for any possible influence these factors may be having on the results. The children with comorbid ADHD in the TS group were excluded in follow-up analyses to show the results for the pure TS group.

### Ethics statement

Children 12 years and older and their parents gave informed written consent prior to inclusion. Children under the age of 12 gave verbal consent prior to being allowed to participate in the study, and the parents of these children provided informed written consent for their children to participate. The study was conducted in accordance with the Helsinki Declaration of the World Medical Association Assembly. It was approved by the Regional Committee for Medical Research Ethics in Eastern Norway (REK-Øst), and by the Privacy protection ombudsman for research at Innlandet Hospital Trust.

## Results

### Measures of focused attention, core cognitive control and decision-making

The mixed ANOVAs examining performance on the focused attention task over time revealed a significant interaction effect of group x time (F(2, 84) = 3.1, *p* = .048, *η*_*p*_^*2*^ = .07), and a significant main effect for group (F(2, 84) = 8.4, *p* < .*001*, *η*_*p*_^*2*^ = .17) (Table 2). Repeated measures ANOVAs conducted for the groups individually indicated improved focused attention across time for the TDC only (F(1, 48) = 5.4, *p* < .*024*, *η*_*p*_^*2*^ = .10). Separate ANOVAs with Bonferroni correction conducted at T1 and T2 demonstrated that children with ADHD-C had reduced focused attention compared with both the TS and the TDC at T1 and at T2. Even when controlling for the possible confounding effects of FSIQ in the mixed ANOVAs, there was no significant interaction effect of time x FSIQ on the focused attention task.

**Table 2:** Measures of executive functioning (raw scores) at T1 and T2: means and standard deviations within the TS, ADHD-C and TDC groups, and results from Mixed Model ANOVA.

Variable	TS (*n = 19*)	ADHD-C (*n = 33*)	TDC (*n = 50*)	Group	Time	Group x Time ^e)^	TS (*n = 19*)	ADHD-C (*n = 33*)	TDC (*n = 50*)	Group	Time	Group x Time ^e)^	TS (*n = 19*)
	T1	T2	T1	T2	T1	T2	F	p	F	P	F	P	η_p_^2^
Focused Attn. ^a)^	23.2 (19)	22.2 (22)	31.4 (20)	40.8 (38)	19.8 (18)	14.1 (15)	(2,84) 8.45	<.001	.02	ns	3.2	<.05	.07
Core Cognitive Control ^b)^	-1.8 (2.9)	0.1 (1.9)	-2.2 (2.8)	-0.3 (2.5)	-0.3 (2.2)	1.2 (2.1)	(2,93) 6.1	<.01	122.5	<.001	1.1	Ns	
Safer choices ^c)^	12.5 (7.9)	21.5 (16.1)	12.8 (7.9)	12.3 (4.9)	14.1 (9.5)	12.9 (8.6)	(2,94) 2.35	ns	3.4	ns	5.1	<.01	.10
Riskier choices ^d)^	22.7 (12.5)	15.6 (9.0)	19.4 (10.9)	18.5 (11.0)	17.6 (8.6)	19.0 (9.4)	(2,943) .14	ns	2.1	ns	2.5	Ns	

^a)^ Higher values indicate more problems with focused attention,

^b)^ Higher values indicate better cognitive control,

^c)^ The Hungry Donkey Task—Lower level of loss and gain last 4 blocks,

^d)^ The Hungry Donkey Task—Higher level of loss and gain last 4 blocks.

^e)^ Effect size is specified only for significant interactions.

**Fig 1:**
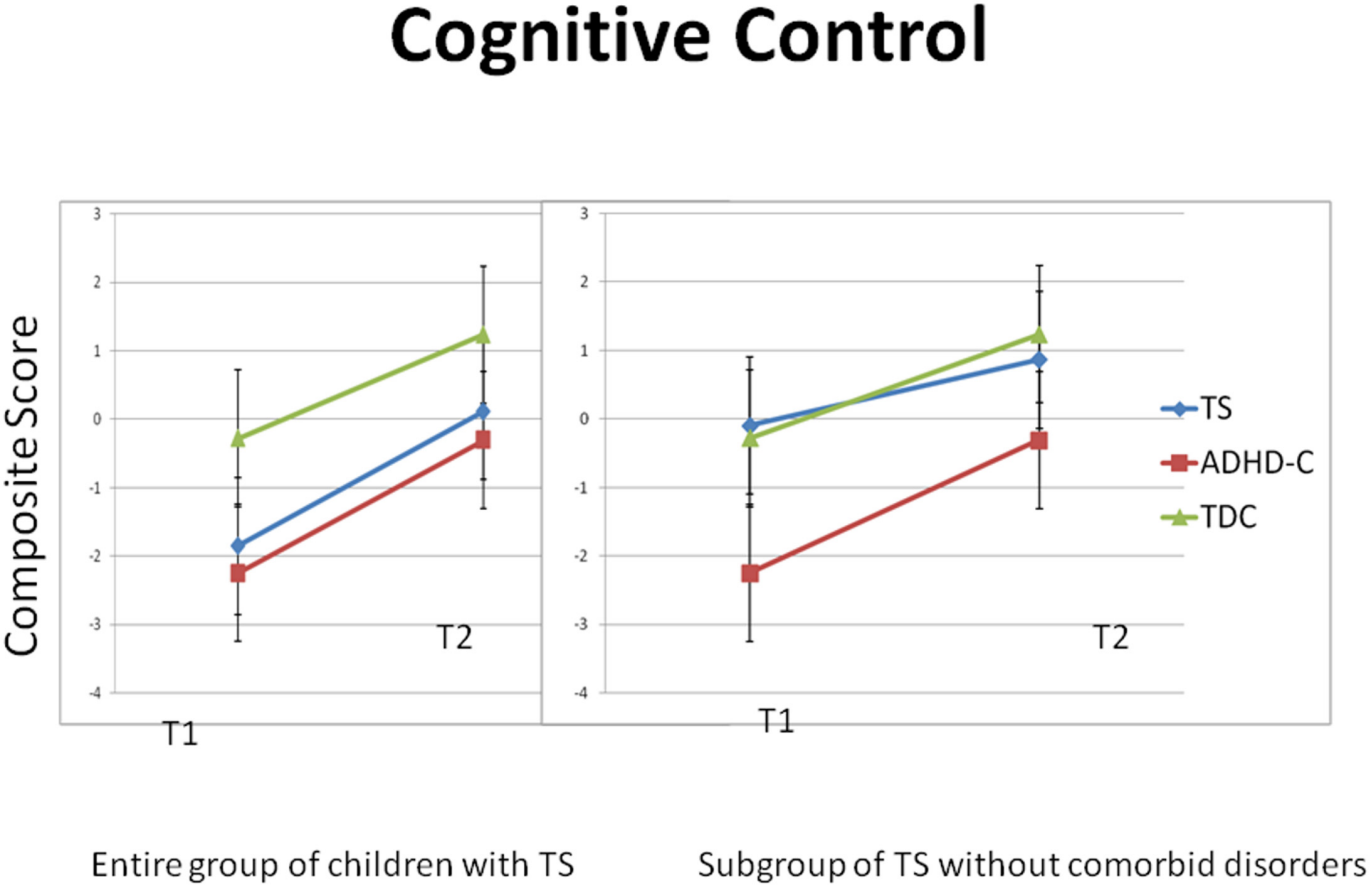
Mean composite score for core cognitive control (working memory, inhibition and mental flexibility) at T1 and T2. The figure to the left includes the entire group of children with TS (19 children), whereas the figure to the right includes only the children with TS-pure (8 children). Vertical bars denote 95% confidence intervals. TS = Tourette’s Syndrome, ADHD-C = Attention-Deficit/Hyperactivity Disorder–Combined subtype, TDC = Typically Developing Children.

Mixed ANOVAs were conducted for the composite score representing core cognitive control, without an interaction effect of group x time for core cognitive control. However, we registered a main effect of time for the composite measure of cognitive control F(1, 93) = 122.5, *p* < .*001*, η_p_^2^ = .57) and a significant main group effect for core cognitive control F(2, 93) = 6.2, *p* < .*01*, η_p_^2^ = .12) (Table 2). Repeated measures ANOVAs conducted for the groups individually indicated significantly improved performance after two years in core cognitive control for all three groups. ANOVAs conducted separately at T1 and T2 with the entire group of children with TS indicated that both the children with TS and the children with ADHD-C scored lower in core cognitive control compared with the TDC at T1 and T2. A follow-up ANOVA including only the children with TS-pure revealed that there was no difference in core cognitive control performance in the children with TS and the TDC. However, the children with ADHD-C performed significantly lower than both the children with TS and the TDC (Table 3 and Fig 1). When controlling for the possible confounding effects of FSIQ in the mixed ANOVAs, there was no significant interaction effect of time x FSIQ on the core cognitive control measure.

**Table 3:** Measures of executive functioning at T1 and T2 (means and SD).

Variable	TS-pure (*n* = 8)		TS group (*n* = 19)		ADHD-C (*n* = 33)		TDC (*n* = 50)	
	T1	T2	T1	T2	T1	T2	T1	T2
Focused attention ^a)^	28 (19.8)	16 (14.9)	23 (18.9)	22 (22.3)	33 (20.9)	41 (36.9)	20 (18.3)	14 (17.2)
Core cognitive control ^b)^	-0.1 (2.7)	0.1 (3.2)	-1.8 (2.9)	-0.2 (2.2)	-2.7 (3.1)	-0.5 (2.6)	-0.3 (2.2)	1.2 (2.1)
Safer choices ^c)^	11 (6.5)	20 (12.0)	12 (7.9)	21 (15.7)	13 (7.8)	12 (4.9)	14 (9.5)	13 (8.6)
Riskier choices ^c)^	23 (9.6)	13 (5.4)	23 (12.5)	16 (8.8)	20 (10.8)	18 (10.7)	18 (8.6)	19 (9.4)

^a)^ Higher values indicate more problems with focused attention.

^b)^ Higher value indicates a better cognitive control

^c)^ Higher values indicate a higher rate of selection.

**Fig 2:**
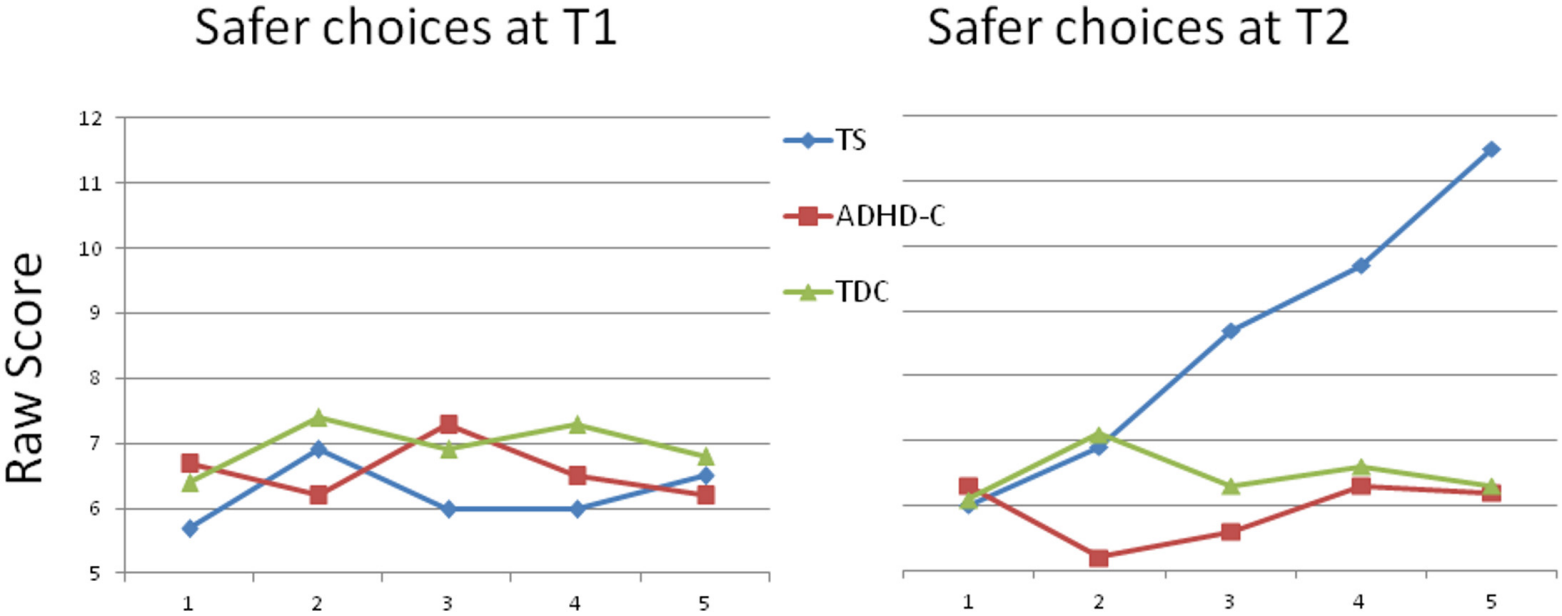
Rates of selecting an advantageous, less risky alternative, across 5 blocks of trials of the HDT at T1 and T2. The ‘safer’ choice tendency in the TS group at T2 involves a preference for frequent, lower-level losses compared with a less frequent higher-level loss alternative.

The mixed ANOVAs examining performance on the measures for safer and riskier choices revealed no significant interaction effect of group x time, nor main effect of time or group. A significant interaction effect of group x time, however, was registered for safer choices (F(2, 94) = 5.1, *p* = .008, η_p_^2^ = .10), but no main effect of time or group for this measure (Table 2, Fig 2). Interestingly, repeated measures ANOVAs conducted for the groups individually did not reveal any significant change for any of the groups over time. Separate post-hoc comparisons at T1 and T2, however, indicated that the TS group at T2 preferred the safer choice option significantly more often compared with the ADHD-C (*p* = .002) and the TDC (*p* = .002). When controlling for the possible confounding effects of FSIQ in the mixed ANOVAs, there was no significant interaction effect of time x FSIQ on the decision-making measures. (Results for measures of executive functioning over time are presented in Table 2.)

Eta squared indicated a moderate effect size for focused attention (η_p_^2^ = .07) and for Safer choices (η_p_^2^ = .10), but a small effect size for the cognitive control and decision-making measures (η_p_^2^ = .01-.03) [86].

### Emotional control problems and symptoms of anxiety and depression

The mixed ANOVAs examining parent-report of emotional control problems revealed a significant interaction effect of group x time for this measure (F(2, 97) = 3.2, *p* = .043, η_p_^2^ = .06). There was a main effect for time (F(2, 97) = 7.6, *p* = .007, η_p_^2^ = .07), and a significant main effect for group (F(2, 97) = 49.8, *p* < .*001*, η_p_^2^ = .51) (Table 4). Repeated measures ANOVAs conducted for the groups individually indicated a reduction of emotional control problems for the children with TS only (F(1, 17) = 8.5, *p* < .*01*, *η*_*p*_^*2*^ = .33). Separate post-hoc comparisons at T1 and T2 indicated that the parents of both children with TS and those with ADHD-C reported significantly more emotional control problems in their children compared with the TDC at both points in time. When controlling for the possible confounding effects of motor tics, phonic tics and ADHD symptoms, we found a significant interaction effect of time x ADHD symptoms only (F(1, 94) = 6.98, *p* = .*01*, η_p_^2^ = .07).

**Table 4:** Measures of emotional control problems and symptoms of anxiety and depression at T1 and T2 (raw scores): means and standard deviations within the TS, ADHD-C and TDC groups, and results from Mixed Model ANOVA.

	TS (*n = 19*)		ADHD-C (*n = 33*)		TDC (*n = 50*)		Group		Time		Time x Group ^d)^		
Variable	T1	T2	T1	T2	T1	T2	F	p	F	P	F	p	η_p_^2^
Emotional control problems ^a)^	65.4 (12.8)	59.9 (15.1)	61.1 (15.9)	58.2 (14.7)	41.0 (4.3)	41.3 (4.7)	(2, 97) 49.8	<.001	7.58	<.01	3.35	<.05	.07
Anxiety symptoms ^b)^	13.7 (9.1)	10.8 (9.7)	16.1 (9.7)	13.4 (8.2)	5.7 (4.3)	5.2 (5.5)	(2, 94) 24.0	<.001	5.23	<.05	1.0	ns	
Depression symptoms ^c)^	4.6 (4.5)	4.7 (5.9)	7.0 (4.4)	6.7 (4.6)	2.2 (2.3)	2.1 (2.4)	(2,93) 26.9	<.001	.01	Ns	.04	ns	

Note. TDC; Typically Developing Children,

^a)^The Behavior Rating Inventory of Executive Function (parent-report)—Emotional Control scale,

^b)^ The Revised Children’s Manifest Anxiety Scale, second edition (self-report). The total raw score is reported, which is based on 40 questions relating to physiological anxiety, worry and social anxiety. For the age group 9–14 years, a total score of 24+ would be considered in the clinical range (Moderately problematic) [87].

^c)^ The Short Mood and Feelings Questionnaire (self-report). The raw score is reported. The clinical cutoff is set at 11; a higher score signifies depression [76].

^d)^ The effect size is specified only for significant interactions.

**Fig 3:**
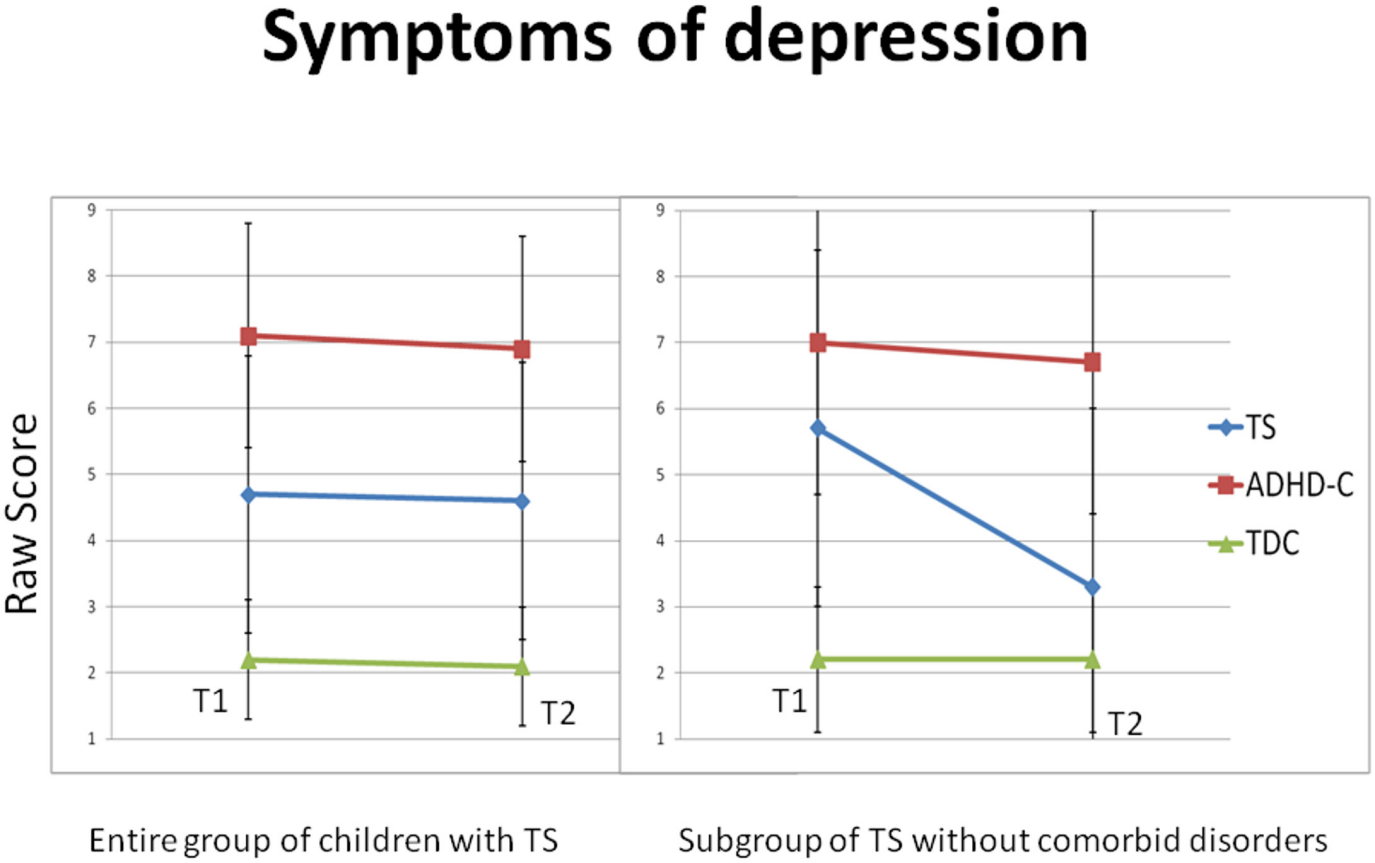
Mean raw scores for symptoms of depression over a two year period during childhood and adolescence. The figure to the left includes the entire group of children with TS (19 children), whereas the figure to the right includes only the children with TS-pure (8 children).Vertical bars denote 95% confidence intervals. TS = Tourette’s Syndrome, ADHD-C = Attention-Deficit/Hyperactivity Disorder–Combined subtype, TDC = Typically Developing Children.

The mixed ANOVAs examining the self-report of symptoms of anxiety and depression revealed no interaction effect of group x time on either self-report measure (Table 4). For symptoms of anxiety, we found a main effect for time (F(2, 94) = 5.2, *p* = .02, η_p_^2^ = .05) and group (F(2, 94) = 24.0, *p* < .001, η_p_^2^ = .34). Repeated measures ANOVAs conducted for the groups individually did not reveal any significant change for any of the groups over time. Separate ANOVAs conducted at T1 and T2 indicated that both the children with TS and the children with ADHD-C reported significantly more anxiety symptoms compared with the TDC at both T1 and at T2. When controlling for the possible confounding effects of motor tics, phonic tics and ADHD symptoms for the anxiety measure, only a significant interaction effect of time x motor tics was present (F(1, 91) = 5.7, *p* < .*02*, η_p_^2^ = .06). For symptoms of depression as dependent variable, only a main effect for group (F(2, 93) = 27.0, *p* < .*001*, η_p_^2^ = .37) was significant. Repeated measures ANOVAs conducted for the groups individually did not reveal any significant change for any of the groups over time. Separate ANOVAs conducted at T1 and T2 showed that the children with ADHD-C reported significantly more depression symptoms than the children with TS, who reported significantly more depression symptoms than the TDC at T1 and at T2 (Fig 3). Follow-up ANOVAs including only the children with TS-pure (N = 8) revealed that these children at T1 reported significantly more depression symptoms compared with the TDC. They showed a similar high level of depression symptoms as the children with ADHD-C. At T2, the children with TS-pure reported no difference in depression symptoms compared with the TDC and significantly less depression symptoms than the children with ADHD-C (Table 5 and Fig 3). When controlling for the possible confounding effects of motor tics, phonic tics and ADHD symptoms for the anxiety measure, no interaction effect was significant for any of the measures. (Results for measures of emotional control problems and symptoms of anxiety and depression over time are presented in Table 4.)

**Table 5:** Emotional control problems and symptoms of anxiety and depression (raw scores) at T1 and T2 (means and SD). Higher values indicate higher symptom load.

Variable	TS-pure (*n* = 8)		TS group (*n* = 19)		ADHD-C (*n* = 33)		TDC (*n* = 50)	
	T1	T2	T1	T2	T1	T2	T1	T2
Emotional control problems ^a)^	63 (16.1)	58 (14.3)	65 (12.8)	60 (15.1)	61 (15.7)	58 (14.7)	41 (4.3)	41 (4.7)
Anxiety symptoms ^b)^	16 (9.9)	8 (4.4)	14 (8.9)	11 (9.7)	15 (9.7)	13 (8.2)	6 (4.3)	5 (5.4)
Depression symptoms ^c)^	6 (5.7)	3 (3.6)	5 (4.3)	5 (5.8)	7 (4.6)	7 (4.6)	2 (2.3)	2 (2.4)

^a)^ The Behavior Rating Inventory of Executive Function (parent-report)–Emotional Control scale,

^b)^The Revised Children’s Manifest Anxiety Scale, second edition (self-report),

^c)^The Short Mood and Feelings Questionnaire (self-report).

Non-parametric analyses applying the Kruskal-Wallis test with an alpha level of .01 were conducted for the analyses relating to symptoms (emotional control problems, depression symptoms, and anxiety symptoms) at both T1 and T2 to account for any possible skewness in distributions. These analyses support the findings of group differences as described in this section and in Table 3.

We conducted independent-samples t-tests comparing symptoms at T2 for the children in the clinical groups taking psychostimulant medication to account for the potential influence of psychostimulant medication on emotional symptoms for the interim period between T1 and T2 (Table 6). No significant difference in emotional control problems was revealed between those taking psychostimulant medication *(M* = 62.7, *SD* = 11.8) and those not taking psychostimulant medication (*M* = 55.7, *SD* = 16.2; *t* (47) = 1.69, *p* = .10.) No significant difference in anxiety symptoms was reported by those on psychostimulant medication *(M* = 15.1, *SD = 8*.*9)* compared with those not taking psychostimulant medication (*M* = 10.4, *SD* 8.6; *t*(44) = 1.79, *p* = .08). Children on psychostimulant medication (*M* = 7.35, *SD* = 5.88) compared with those not taking psychostimulant medication *(M* = 5.00, *SD* = 4.62; *t*(44) = 1.52, *p* = .14.) did not differ with respect to self-reported symptoms of depression. All analyses were also conducted excluding the one child with ADHD-C who had forgotten to not take psychostimulant medication 24 hours before the assessment at T2. Excluding this single participant did not show any effect on any of the results reported above.

**Table 6:** ADHD symptoms (raw scores) at T1 and T2 for three clinical groups (means and SD).

Variable ^a)^	TS-pure (*n* = 8)		TS group (*n* = 19)		ADHD-C (*n* = 33)	
	T1	T2	T1	T2	T1	T2
Inattention ^b)^	8.7 (7)	9.0 (5)	13.7 (6)	9.2 (5.2)	16.6 (5.8)	12.8 (7)
Hyperactivity ^b)^	10.5 (8)	9.9 (7)	11.9 (7)	7.6 (6)	13.6 (6)	8.5 (7)
Total ^b)^	19.2 (14)	16.6 (10)	25.6 (14)	15.9 (10)	29.9 (9.8)	21 (12)

^*a)*^ To assess disorder severity in the clinical groups, we provide results for symptoms of inattention and hyperactivity for the children with TS including those with comorbidities (i.e. TS group), the children with TS excluding those with comorbidities (i.e. TS-pure) and the children with ADHD-C.

^*b)*^ ADHD Rating Scale IV. Higher values indicate higher symptom load.

### Associations among changes in performance from T1 to T2 on measures of cognitive control and decision-making with symptoms of anxiety and depression and emotional control problems

Results for the TS group showed a significant relationship between safer decision-making and less problems with emotional control reported by parents (*r* = .72). When we did the same analysis excluding the children with comorbidity in the TS group, the relationship between safer decision-making and less emotional control problems increased (*r* = .93). In analyses conducted in the entire TS group, we found a significant relationship between poorer attention and an increase in anxiety (*r* = .79). However, the latter relationship was no longer significant when we excluded the children with comorbidity in the TS group (i.e. the pure TS subgroup). Changes in performance on the neuropsychological tasks and changes in the self- and parent ratings of emotional symptoms and emotional control problems for the ADHD-C or TDC groups were not significantly associated (at p < .01).

Table 6 provides a specification of ADHD symptoms in the clinical groups.

## Discussion

Our results indicate that youth with TS, ADHD-C and TDC develop similarly over two years on core measures of cognitive control. Further, our results showed that children with TS develop a preference for more cautious decision-making compared with the children with ADHD-C and the TDC. Different brain networks underlie various aspects of cognition and are thought to mature at different rates [13, 88]. Our results provide evidence that core cognitive control functions develop continuously throughout adolescence, whereas focused attention and aspects of decision-making do not follow the same developmental trajectory. We found a difference in performance at T1 and T2 between the TDC and the children with TS and ADHD-C on a composite measure of core cognitive control, but the results for the children with TS depended on whether those with comorbid disorders were included in the analyses or not. When children with TS and comorbid disorders were included in the analyses, the performance of those children was more similar to the children with ADHD-C, whereas if the children with TS and comorbid disorders were excluded in the analyses, the performance of the TS group was more similar to the TDC. However, no significant group difference in maturational change in cognitive control after two years could be detected when comparing children with TS, ADHD-C or TDC. The change across two years was similar in the TS pure group and the TS group as a whole. However, findings derived in the focused attention task indicated a maturational improvement in the TDC, but not in the children with TS or with ADHD-C. The analyses including only the children with TS-pure showed a similar rate of maturational development as the TDC on the focused attention task. These results provide further evidence of the potential influence of comorbidities on cognitive development in children with TS.

Accounting for the influence of comorbidity and overlapping symptoms is important when carrying out research involving children with TS [89]. A complex interplay of factors may underlie attention problems [90]. Although the general level of inattention and hyperactivity symptoms is lower in the TS-pure group compared with the TS group as a whole at T1 and T2, both groups show a lower level of overall ADHD symptoms compared with the children with ADHD-C at both T1 and T2. Interestingly, the level of excessive motor activity symptoms is similar in all three clinical groups at T2. The general level of inattention symptoms seems to be the factor discriminating between the children with TS and the children with ADHD and not hyperactivity/impulsivity. When we examined the results of the decision-making measures indicating a safer or riskier response style in selecting between options that both gave an overall favorable outcome, a distinct pattern emerged in the group’s responses. We expected that children with ADHD-C would prefer the riskier choice. Although the mean for the group with ADHD-C did suggest a tendency, the result was not significant. On the other hand, toward the end of the task at T2, the children with TS clearly preferred the safer choice to the riskier choice. The tendency to prefer the safer choice was more prominent in the TS group when we excluded the children with comorbid ADHD from the group. Dissociable neural responses in reward systems for decision-making tasks are well established [91], and may be playing a role in differentiating response tendencies in the two clinical groups. Both the children with TS and the children with ADHD-C were presumably subconsciously learning which doors gave overall favorable outcomes. However, the TS group clearly favored the safer choice, whereas the ADHD-C group chose differently. One interpretation of this distinct pattern is that children with TS are *more* sensitive to large penalties and thus subconsciously tend to avoid large losses, whereas ADHD-C are *less* sensitive to large penalties and are thus more inclined to opt for a riskier strategy. Sensitivity to penalties has been shown to be related to dopamine transmission, which seems dysfunctional in both TS [92] and in ADHD [93, 94]. A subject of future research would be to disentangle the role dopamine levels have on decision-making preferences among risk and reward contingencies for children with TS or with ADHD.

Children with TS, as well as those with ADHD-C, were more rigid in their decision-making response tendencies at T2 than the TDC. Moreover, children with TS and ADHD-C had opposing preferences, whereas the TDC were more balanced in their selection between the two overall advantageous outcomes. The tendency in the ADHD-C group toward a riskier strategy (i.e. less sensitive to magnitude of penalty) is consistent with the findings in a cross-sectional decision-making study involving ADHD children ages 7–12 years compared with TDC [37]. This type of response tendency has not been previously studied in a longitudinal perspective in children with TS. As the brain regions responsible for rational decision-making are not fully developed until early adulthood [29], the children in the current study were presumably not consciously aware of any strategy in their responses. Although we did not specifically question the participants about their decision-making strategy, some underlying factor may have been subconsciously influencing the TS group’s response preferences. The repeated suppression of tics is a subconscious process known to influence their neurobiology [95, 96], and possibly also their decision-making. The change in response pattern in the TS group from T1 to T2 may indicate the emergence of compensatory mechanisms shaping biological functions into tendencies influencing behavior [97, 98]. The overly inhibitory response style emerging through the constant suppression of tics in the TS group could be influencing children with TS to select the less emotionally activating and less impulsive alternative in the decision-making task. For children with TS, an overly cautious response style is particularly activated when the outcome of a response is uncertain [99], as in the decision-making task used in the current study. Youth with ADHD-C, on the other hand, presumably have lower levels of inhibitory capacity, and may thus be less sensitive than young people with TS to the choice involving a high loss.

As expected, the young individuals with TS or ADHD-C reported more symptoms of anxiety and depression than the TDC at T1, but their self-report of anxiety and depression provided an unexpected result at T2. Whereas both the children with TS and the children with ADHD-C self-reported significantly more symptoms of anxiety at T2 compared with the TDC, the results for symptoms of depression was more complex. The ADHD-C group had significantly more symptoms of depression than both the children with TS and the TDC at T1 and T2, and the children with TS had significantly more depression symptoms than the TDC at both T1 and T2. However, when performing new analyses including only the children with TS but without comorbidities, the level of depression symptoms for this subgroup of children with TS at T2 was still significantly lower than the ADHD-C group, but no longer significantly higher compared with the TDC. Many problems in TS are attributed to comorbidities, but a study examining the impact of ADHD on TS concluded that TS was associated with significant internalizing problems (e.g. anxiety and depression) in adolescence, regardless of ADHD comorbidity [100]. The results from our study indicate that an ADHD-C diagnosis may be associated with more symptoms of depression than a TS diagnosis over time. Importantly, however, both clinical groups reported a significantly higher level of anxiety and depression symptoms compared with TDC at T1, indicating that symptoms of depression were also a problem for the children with TS regardless of ADHD comorbidity. The general high level of internalizing symptoms for both groups has clinical relevance, and may suggest that the treatment received during the two years was more focused on their behavioral problems rather than their perception of well-being. Alternatively, their subjective emotional difficulties could have been more resistant to treatment. A third possibility is that interventions aimed at enhancing their emotional well-being were not offered due to the scarcity of treatment options addressing emotional dysregulation in children with neuropsychiatric disorders.

Increasing self-control over thoughts, emotions and behavior is an important ability developing throughout childhood and adolescence, and parents are in a unique position to follow their child’s development in everyday life during this period. Youth with TS or ADHD frequently display problems self-regulating their actions and responses, which are closely associated with risk-taking activities [48, 101]. Our findings indicate that all three groups improved in their emotional control abilities after two years. Nevertheless, at T2, both clinical groups were still rated by their parents as exhibiting significant emotional control problems compared with the TDC. Considering that 42% of the TS group no longer had motor or phonic tics sufficient to fulfill the criteria for a TS diagnosis at T2, it is interesting to note that the TS group still self-reported significant problems regulating their emotions. Despite a general improvement of tics by age 18 years for most children with TS, impaired psychosocial functioning has been shown to continue into young adulthood [102]. The remission of tic symptoms in many of the youth with TS in our study does not seem to have resulted in a reduction of either parent-reported problems with emotional control or self-reported symptoms of anxiety or depression compared with the TDC. Thus, our findings provide further evidence to support the position that reduced emotional control and symptoms of anxiety and depression may be sources of disability in youth with TS regardless of the presence of active tic symptoms.

For the children with TS, we found a clear relationship between a tendency to select a safer choice on the decision-making task and less emotional control problems. This close relationship was shown to be even stronger when we excluded the children with TS and comorbidities from the analyses. No similar relationship was detected for the ADHD-C group or for the TDC. The finding for the TS group alone seems to suggest that developmental processes promoting more safer decision-making in this group may have a positive impact on their emotional control problems as well. Future studies could examine the relationship between decision-making and everyday emotional control in children with TS, with the aim of finding common underlying mechanisms that could be targeted for interventions.

## Conclusion

All three groups improved similarly in performance in core cognitive control (working memory, inhibition and mental flexibility) over a period of two years in adolescence, but the results for focused attention and decision-making preferences after two years differed among the groups. Comorbidity in the children with TS influenced cognitive performance and symptom severity at baseline and over time. The findings for the TS group regarding the close relationship between safer decision-making and less emotional control problems over time may be related to the emergence of compensatory mechanisms arising from frequent tic suppression and the potential impact this may have on other behaviors. Future studies could be designed to try to disentangle the direction of influence between decision-making and emotions and the balance between top-down and bottom-up dominance at different age groups in youth with TS or with ADHD-C.

### Clinical implications

Based on our results indicating no clear association between improved core cognitive control skills and a reduction in emotional control problems or symptoms of anxiety or depression, it is reasonable to suggest that training core cognitive control skills (e.g. working memory training) may not be a particularly effective approach to treating the emotional difficulties associated with a TS or ADHD-C diagnosis in childhood. Importantly, the persistent high self-report of anxiety and depression symptoms in the children and adolescents with TS or ADHD-C indicates that this should be an important area of focus in treating these two neurodevelopmental disorders. Furthermore, understanding the emerging tendency of children with TS to respond more cautiously—perhaps even in a rigid fashion—compared with children with ADHD-C and TDC may provide clues to underlying mechanisms influencing emotionally salient behavior in children with TS. Results from this study indicate that children with TS and children with ADHD-C may differ in their sensitivity to reinforcement contingencies. This finding is important in that applying reinforcement contingencies is one way parents and therapists can influence behavior in children and adolescents with behavior difficulties [31, 103, 104].

### Strengths and limitations of the study

Among the strengths of the current study are including a set of different informants and perspectives, both self-report and parent-ratings and direct measures of performance, as well as the two-year follow-up design of the same participants from T1 to T2. The wide age range of the sample is a limitation, even though the age distribution is similar for all three groups. An important limitation is the relatively small number of children with TS in the study and the high level of comorbidity; however, the level of comorbidity is similar to typical clinical samples of this population [48]. Understanding the cognitive performance and development of children with TS with and without comorbidities is important for the clinician working with these children. We controlled for the use of psychostimulant interventions during the two-year follow-up period by providing analyses comparing those children receiving medication with those not receiving medication, but we did not have detailed information about other treatment interventions during this period. Future studies should attempt to disentangle the influence of age and other factors by narrowing the age groups investigated and ensuring better control over factors potentially influencing maturational development.
